# Carbon Nanofiber—Sodium Alginate Composite Aerogels Loaded with Vitamin D: The Cytotoxic and Apoptotic Effects on Colon Cancer Cells

**DOI:** 10.3390/gels9070561

**Published:** 2023-07-10

**Authors:** Ozlem Bingol Ozakpinar, Havva Dastan, Merve Gurboga, Fatih Serdar Sayin, Derya Ozsavci, Elif Caliskan Salihi

**Affiliations:** 1 Department of Biochemistry, Faculty of Pharmacy, Marmara University, 34854 Istanbul, Turkey; deryaozsavci@hotmail.com; 2 Department of Biochemistry, Health Sciences Institute, Marmara University, 34865 Istanbul, Turkey; havvadastan60@gmail.com (H.D.); mervegurboga@gmail.com (M.G.); 3 Department of Electrical-Electronics Engineering, Faculty of Technology, Marmara University, 34840 Istanbul, Turkey; fatih.sayin@marmara.edu.tr; 4 Department of Basic Pharmaceutical Sciences, Faculty of Pharmacy, Marmara University, 34854 Istanbul, Turkey; caliskanelif@gmail.com

**Keywords:** vitamin D, drug delivery system, carbon nanofiber, aerogel, colon cancer cells, apoptosis

## Abstract

Colorectal cancer (CRC) is the fourth most commonly diagnosed cancer and the third leading cause of cancer-related deaths worldwide. A substantial body of literature supports the crucial role of vitamin D (VD) in the etiology, progression, prognosis, and treatment of cancer. Recent clinical studies have found an inverse correlation between CRC incidence and serum VD levels. However, the low water solubility of VD and its anticarcinogenic activity at supraphysiological plasma levels, which causes hypercalcemia, required carrier systems. Carbon-based nanomaterials are excellent eco-friendly candidates, with exceptional chemical resistance, efficient mechanical properties, and negligible weight. Furthermore, composite aerogels manufactured from these nanomaterials have gained interest due to their extensive surface areas and porous structures, which make them suitable for delivering drugs. Our research aimed to study the development of composite aerogels loaded with VD by utilizing carbon nanofibers (CNFs) in an aerogel matrix provided to colon cancer cells. For this purpose, Aero1 as a drug delivery system was first prepared and characterized using XRD, FTIR, and SEM methods. Biochemical methods were employed to evaluate the antiproliferative, apoptotic, and anti-migratory effects on colon cancer cells. FTIR and XRD measurements confirmed the production of aerogels. SEM analysis revealed that aerogels have a non-uniform surface. The findings showed that aerogels can effectively deliver VD to the colon cancer cells, while also inhibiting cancer cell proliferation and migration. This research suggests that the Aero1 drug delivery system could be a valuable tool in the fight against colon cancer and other health issues.

## 1. Introduction

Colorectal cancer (CRC) is the third most common cancer worldwide, resulting in 1.8 million cases and ~860,000 deaths each year [1]. Notably, the incidence of CRC continues to rise steadily worldwide, particularly in developing countries that are increasingly adopting a “western” lifestyle. As with other types of cancer, traditional treatments such as surgery, chemotherapy, and radiotherapy used in the treatment of CRC have certain side effects and limitations. Due to poor chemical and physical properties, low bioavailability, and poor tissue selectivity of chemotherapeutic drugs, patients may suffer from many serious side effects [2,3]. Radiotherapy can cause severe DNA damage in patients susceptible to radiation damage, leading to the progression of tumorigenesis [4]. Immunotherapy, which has been applied in recent years, has limitations such as the high cost of the drugs, the development of drug resistance, and many side effects [5,6]. The elucidation of the molecular mechanisms underlying the pathogenesis, metastasis, and drug resistance development in CRC, which is caused by multifactorial processes, provides the emergence of new alternative therapies, such as miRNA replacement therapy [7], which has shown promising results, especially when combined with chemotherapeutic agents and nanoparticle-based drug carriers for targeted therapy [8]. 

Clinical studies have revealed that ecological variation in vitamin D (VD) levels between populations is an environmental factor contributing to the variation in CRC incidence [9,10,11]. VD is a fat-soluble hormone synthesized through the photolytic activation of UV-B radiation on the 7-dehydrocholesterol molecule present in the epithelial tissue. This activation results in the formation of pre-vitamin D3, which is subsequently absorbed and metabolized in the liver to form 25-hydroxyvitamin D3. The latter subsequently reaches the renal tubules and is converted into the active form, 1,25-dihydroxyvitamin D3 [11]. 

VD is a prohormone that regulates a wide range of physiological processes. It has been identified as the most effective hormone in the regulation of intracellular and extracellular calcium and phosphorus. Since Garland’s hypothesis in the early 1980s suggested the possible anticancer activity of VD [12], its anticarcinogenic role has been demonstrated in numerous preclinical and clinical studies [13,14]. However, the anticarcinogenic activity of VD occurs at supraphysiological plasma levels that cause hypercalcemia. Hypercalcemia has various clinical manifestations that affect multiple organ systems, regardless of the underlying cause or etiology [15]. Hypercalcemia, the major toxic effect of VD, has, therefore, greatly limited the use of VD in clinical practice [16]. A targeted drug delivery system with a low calcemic VD effect appears to be the most desirable system in cancer therapy.

Epidemiological studies have also revealed that a significant portion of the world’s population has low levels of VD. The geographical location (altitude and latitude), sun angle and duration of exposure to sunlight, air pollution, low number of foods naturally containing VD, low solubility in gastrointestinal fluids, and poor bioavailability may be suggested as reasons for low VD levels [9]. Notably, the consequences of low VD levels are comprehensive and extend beyond the impairment of calcium homeostasis and skeletal health. Several lines of evidence have implicated VD deficiency in the pathogenesis of numerous diseases, including cancer, autoimmune disorders, diabetes, osteoarthritis, and periodontal diseases [17]. Therefore, it is imperative to address this public health concern and develop effective interventions to mitigate the adverse effects of low VD levels. 

The recognition of VD deficiency as a risk factor for tumorigenesis has led to the development of novel therapies using nanotechnologies that incorporate VD into pharmaceutical formulations without reducing its bioavailability or activity. There are various delivery systems for loading VD for cancer therapy and these systems are mostly focused on polymers [18,19,20]. In this polymer carrier system, which is predominantly developed for breast cancer, it has been reported that the half-life of VD is completed before reaching the target tissue. Furthermore, challenges encountered in polymer preparation, the frequency of polymer administration, and high costs are negative characteristics in terms of clinical utilization.

With the development of nanotechnology, many high-efficiency nano drug delivery systems have been developed, such as lipid nanoparticles [21], polymer nanoparticles [22], and liposomes [23], which can deliver chemotherapeutic drugs, genes, and vaccines for the treatment of CRC. However, most of the potential products focus on liposomes and polymeric materials, ignoring the promising potential of inorganic materials with unique physicochemical properties. Specifically, carbon allotropes such as graphene oxides (GOs), carbon nanotubes (CNTs), and nanodiamonds (NDs) possess similar unique characteristics that make them suitable for use as target-specific drug-loadable nanocarriers in drug delivery applications. Therefore, carbon-based nanomaterials (CBNs) have garnered significant attention owing to their exceptional chemical resistance, favorable mechanical properties, and negligible weight.

Recent literature data has shown that CBNs can act as anticancer agents and, when combined with cytotoxic drugs such as etoposide [24], dexamethasone [25], paclitaxel [26], and cisplatin/carboplatin [27], can sensitize cancer cells to these agents. However, there are some studies showing that these CBNs may be toxic to some normal human cells [28,29]. Therefore, it is still an open question that requires further research on these materials. 

Carbon nanofibers (CNFs) are a class of hollow, highly hydrophobic, and cost-effective CBNs that have recently gained considerable attention in biomedical applications due to their outstanding mechanical, thermal, and electrical properties [30,31]. The unique characteristics of CNFs make them highly attractive for a range of bioengineering applications, including drug delivery, tissue engineering, and biosensing. Particularly, when incorporated into composite biomaterials, CNFs can significantly enhance the water diffusion and mechanical properties of the resulting material [32]. For instance, Gamez et al. (2020) demonstrated that films composed of calcium alginate and CNFs exhibited significant potential as hydrophilic materials for various complex applications in biomedicine and bioengineering [33].

Alginate is a naturally occurring polysaccharide extracted from brown seaweed [34] and is biodegradable, biocompatible, and considered safe for biomedical applications. Alginate-based drug delivery systems have been developed for various drugs, including chemotherapeutic agents for cancer treatment [35]. One of the key advantages of alginate-based drug delivery systems is their ability to target specific tissues or cells. This can be achieved by incorporating targeting molecules into the alginate matrix, such as antibodies or peptides, that bind specifically to cancer cells. By delivering drugs directly to cancer cells, these systems can improve the efficacy of treatment while minimizing side effects.

In this study, we hypothesized that VD-loaded CNFs, incorporated into a sodium alginate matrix, may exhibit anticancer activity on colon cancer cells without causing cytotoxicity in normal human cells, despite the contradictory results observed for other CBNs in the literature. Encapsulation of these CNFs into alginate aerogels or beads may be a suitable strategy to reduce cytotoxicity. Although further research is needed, it has been suggested that aerogel density plays an important role in drug adsorption [36]. Effective results at low doses can be achieved by incorporating multiple drugs or compounds into aerogels with high biocompatibility. In light of these interests, the aim of this study was to develop and optimize targeted, low-cost, and biocompatible VD delivery systems using CNFs as carrier materials and investigate their antiproliferative, apoptotic, and cell migration inhibitory activities on human colorectal cancer cells for the first time.

## 2. Results and Discussion

### 2.1. Characterization of Aerogels

**Figure 1:**
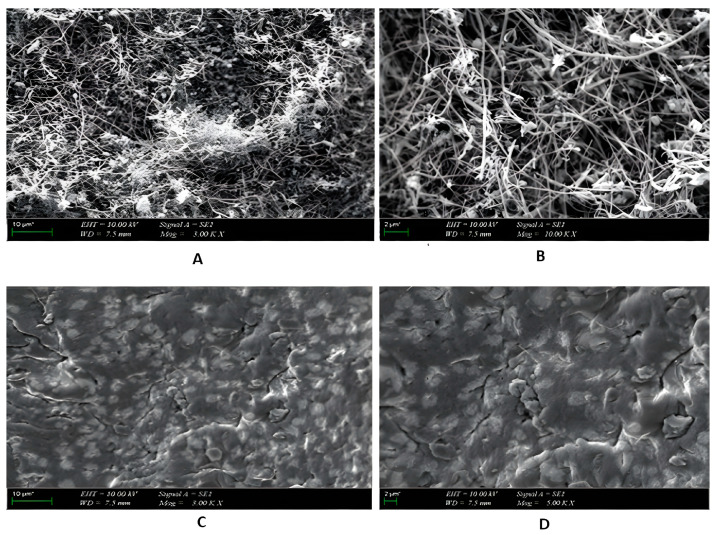
Representative SEM images of the ACNF-VD (**A**,**B**) and Aero1 (**C**,**D**) samples. The scale bar represents 10 µm at (**A**,**C**) and 2 µm at (**B**,**D**).

SEM was used to analyze the surface morphology and structure of the nanofibers and aerogel samples. The fibrous structure, dimensions, and shape of the nanofibers are clearly visible in both SEM images (Figure 1A,B). The CNFs used are approximately 100 nm × 20–200 µm in size on average. CNFs were embedded in the alginate matrix during the fabrication of the composite aerogels seen in Figure 1C,D. Aerogel samples have a non-uniform surface, as seen in the images. 

**Figure 2:**
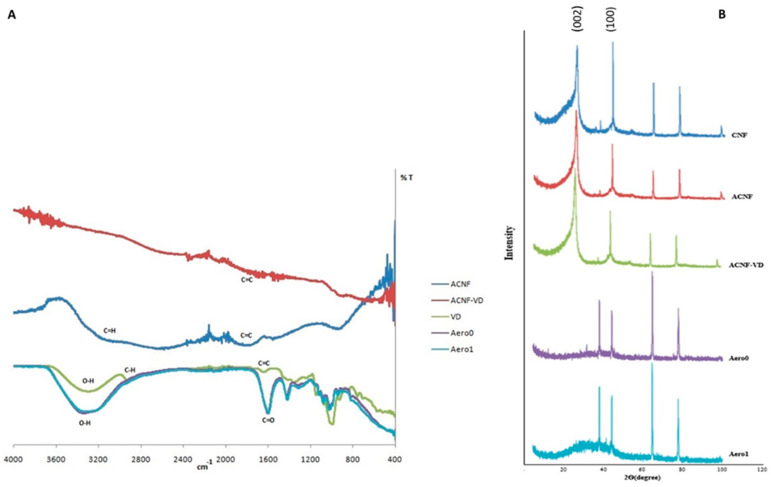
FTIR spectra of VD, ACNF, ACNF-VD, and the aerogels samples (**A**), XRD patterns of CNF, ACNF, ACNF-VD, and the aerogels samples (**B**).

FTIR spectra of VD, ACNF, ACNF-VD, and the aerogel samples are given in Figure 2A. The typical spectrum for VD is seen in the figure, alongside the loaded/unloaded ACNFs and the loaded/unloaded aerogel samples. There are stretching bands showing O-H and C-H bonds around 3300 cm^−1^ and 2900 cm^−1^. There are also several peaks corresponding to C=O and C=C stretching bands at around 1600 cm^−1^ [37]. FTIR spectra of the aerogel samples have absorption bands around 3300 cm^−1^, indicating the O-H stretching of the alginate matrix. Both spectra have similar peaks, and the small variation in peak positions at approximately 3300 cm^−1^ is excluded due to the addition of VD-loaded ACNF to the alginate matrix. There are also small changes in the positions of many peaks between 1600 cm^−1^ and 600 cm^−1^. The small absorption bands at around 1800 cm^−1^ are attributed to C=C bonds belonging to graphene-based materials [38,39,40,41]. There are minor changes in the peak positions in the FTIR spectrum of the ACNF after the loading of VD because the physical adsorption of VD on the ACNF is mainly based on hydrophobic interaction.

X-ray diffraction patterns of CNFs, ACNFs, and the composite aerogel samples were recorded and shown in Figure 2B. The characteristic peaks of the CNFs are seen in the XRD spectra of the CNF and ACNF samples, including the VD-loaded ACNF sample. There are peaks around 2θ of 26 that can be assigned to the (002) plane, indicating the amorphous nature of carbon. There are also peaks around 2θ of 44, 64, and 77, which are also the characteristic peak positions of carbon nanofibers with slight shifts in 2θ values [42]. The reflection peaks around 2θ of 45 correspond to (100) planes of the graphite structure in accordance with the previous studies [43]. Crystal structural changes are clearly observed in the patterns of Aero0 and Aero1 samples after combining them with the alginate matrix to form the composite aerogel. There are broad bands in the diffraction spectrum of alginate-containing composites, indicating the amorphous nature of the alginate matrix. The composite aerogels exhibit a reduced crystalline nature, and the changes in peak positions and shapes demonstrate the evolution of the crystalline composition induced by alginate [44,45,46].

### 2.2. Assessment of Cell Viability of Aerogels

A growing body of literature supports the notion that VD plays a significant role in the onset, progression, prognosis, and treatment of cancer [47,48]. Accumulating evidence from both preclinical and clinical studies strongly suggests that VD deficiency increases the risk of developing CRC [49,50,51]. Therefore, it is increasingly being considered that the prevention of VD deficiency may be an effective, safe, and cost-effective approach to reducing the incidence of CRC and improving prognosis.

1,25-dihydroxyvitamin D3 (1,25(OH)2D3), the biologically active form of VD3, is widely recognized for its influence on bone, calcium, and phosphate homeostasis. However, recent research has revealed its involvement in various tissues expressing the vitamin D receptor (VDR), independent of its effects on bone metabolism [52,53,54]. Despite this, it is important to note that nanomolar doses of 1,25(OH)2D3 are required to elicit these non-classical effects. As physiological serum concentrations of 1,25(OH)2D3 are in the picomolar range, higher doses can lead to hypercalcemia. As a result, analogs of 1,25(OH)2D3 have been developed with the goal of minimizing calcemic side effects while retaining the non-classical functions of 1,25(OH)2D3 [52]. Despite the promising results observed with various VD analogs in animal cancer models, no such analogs have been adopted in clinical practice for the treatment of cancer. This is believed to be due to differences in activity, metabolism, and VDR interactions relative to endogenous forms of VD. Therefore, it is crucial to investigate alternative means of delivering VD to cancer cells, such as utilizing DDS, in order to enhance its therapeutic efficacy. The primary objective of this study is to examine the anticarcinogenic potential of targeted, cost-effective, and biocompatible VD carrier systems utilizing CNF as a carrier material.

In recent years, CNFs have emerged as nanomaterials with exceptional properties and a wide range of potential applications. Studies have demonstrated that CNFs have a lower toxicity profile compared to monolayer and multilayer carbon nanotubes in lung fibroblast cells [55] and mouse keratinocytes [56]. Despite this, research on the use of CNFs in cancer treatment is currently limited [57]. Furthermore, no study has suggested its potential use as a drug carrier for CRC treatment.

The current challenge in cancer therapy is the deleterious impact of cytotoxic drugs on both malignant and healthy cells. Our study investigated the cytotoxic effects of CNFs on NIH3T3 cell proliferation, revealing inhibition rates of 40–60% depending on the dose. Despite the modification of CNFs with acid and glycine (ACNF) and loading VD onto these ACNFs, the cytotoxic activity persisted (data not shown). A literature review revealed that CNFs have been combined with biopolymers, such as alginate, to produce non-cytotoxic composites with improved physical and biological properties, including mechanical strength, thermal stability, wettability, and cellular adhesion and proliferation. Among these composites, aerogels, which have a high surface area and porous structure, are particularly desirable as drug delivery systems. Studies have demonstrated the effectiveness of ibuprofen-loaded silk fibroin aerogels in sustained drug release and transport to the target tissue [58], as well as alginate aerogels in drug transport and release [59].

**Figure 3:**
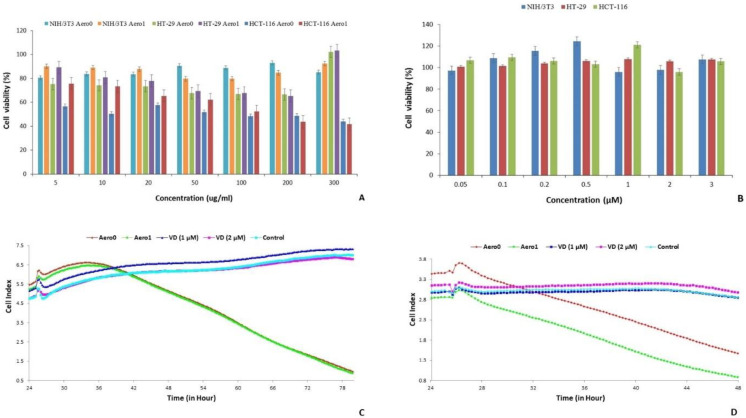
The dose-dependent antiproliferative effects of Aero0 and Aero1 on NIH/3T3, HCT-116, and HT-29 cell proliferation (**A**) and VD (**B**). The effects of Aero0, Aero1 (200 µg/mL), and VD (1 µM and 2 µM) on HT-29 (**C**) and HCT-116 (**D**) cell proliferation.

In this study, the biocompatibility of the aerogel carrier system was evaluated by determining its cytotoxic effects on NIH3T3 cells at concentrations ranging from 50 to 300 µg/mL. The results demonstrated that Aero1, the aerogel delivery system loaded with VD, significantly enhanced the proliferation of NIH3T3 cells. Conversely, Aero0 exhibited inhibitory effects on cell proliferation, particularly at a concentration of 300 µg/mL (Figure 3A). The goal of this research was to develop a DDS that does not exhibit cytotoxicity toward normal cells. Therefore, the effects of Aero1 on HT-29 and HCT-116 colon cancer cell lines were examined and analyzed. To determine the amount of VD in the aerogels, a batch adsorption method was employed. The adsorption capacity was calculated using Equation (1) given below. The adsorption capacity of CNFs for VD was found to be 218.57 mg/g. Subsequently, the VD content of the aerogels was calculated using this capacity value to ensure that the samples contained an equal amount of VD for the cell culture experiments.

The characterization of Aero1 was conducted to investigate whether the observed effect of the system on normal cell proliferation was a result of the ACNF and/or VD. The VD content within the structure of Aero1 was quantified using the batch adsorption method and determined to be 254.29 mg/g. Cytotoxicity and antiproliferative effects of equivalent VD concentrations were examined using the MTT method on normal cells (NIH/3T3) and colon cancer cells (HT-29 and HCT-116) at a dose range of 0.05–3 µM (Figure 3B). The results indicated that the system did not exhibit cytotoxic effects on normal and cancer cells within this dose range. These findings are consistent with previous studies in the literature. Varghese et al. (2020) investigated the cytotoxic effects of VD3 on HCT-116 cells and determined that the IC50 concentration was 15 µM [60]. Razak et al. found that nanoemulsions containing 0.5, 1.0, and 2.0% (*v*/*v*) VD caused inhibition in HCT-116 and HT-29 cells, depending on the dose [61]. Additionally, another study observed that the administration of VD in the form of a nanoemulsion had a cytotoxic effect on HCT-116 and HT-29 cells, with IC50 concentrations of 40.6 µM and 50.1 µM, respectively, after 24 h of treatment [62]. Similary, Almouazen et al. reported that the nanoencapsulated VD induced significant growth inhibition when compared to free VD, and the PLA NPs enhanced the intracellular delivery of VD in breast cancer cells [63]. 

The IC50 concentration of Aero1 was determined to be 141.2 µg/mL in HCT-116 cells and 592 µg/mL in HT-29 cells after 24 h. However, it was not possible to determine the IC50 concentration of Aero0, as the cell proliferation values did not exhibit a linear relationship within the dose range of Aero0. These findings suggest that Aero1 is more effective than Aero0 in a dose-dependent manner. Additionally, the improved dispersibility of Aero1, as observed during the experiments, is suggested to contribute to the linear antiproliferative effect on colon cancer cells.

A XCelligence Real-Time Cell Analysis was also conducted to evaluate the antiproliferative activity of Aero0, VD, and Aero1 on HCT-116 and HT-29 cells. The results of the analysis indicated that the antiproliferative activities of Aero0 and Aero1 on HT-29 cells began around the 45th hour and reached their maximum levels at the end of the 80th hour, as depicted in Figure 3C. In contrast, the antiproliferative activities of Aero0 and Aero1 began around the 30th hour on HCT-116 cells and reached their peak at the end of the 48th hour, as observed in Figure 3D. Furthermore, it was determined that Aero1 exhibited a higher level of antiproliferative activity in HT-29 and HCT-116 cells in a dose-dependent manner compared to Aero0. Additionally, it was established that VD alone did not demonstrate efficacy at doses of 1 µM and 2 µM (Figure 3C,D). 

### 2.3. Apoptotic Potential of Aerogels

The inhibition of VD-mediated proliferation in cancer cells is achieved through the control of cell death mechanisms, such as apoptosis [64,65]. In our study, we also investigated the apoptotic effect of aerogels on colon cancer cells using biochemical and morphological methods. Since caspase signals play a vital role in the process of cell apoptosis, the activities of caspase 3, 8, and 9 enzymes were determined using the spectrophotometric method in this study.

**Figure 4:**
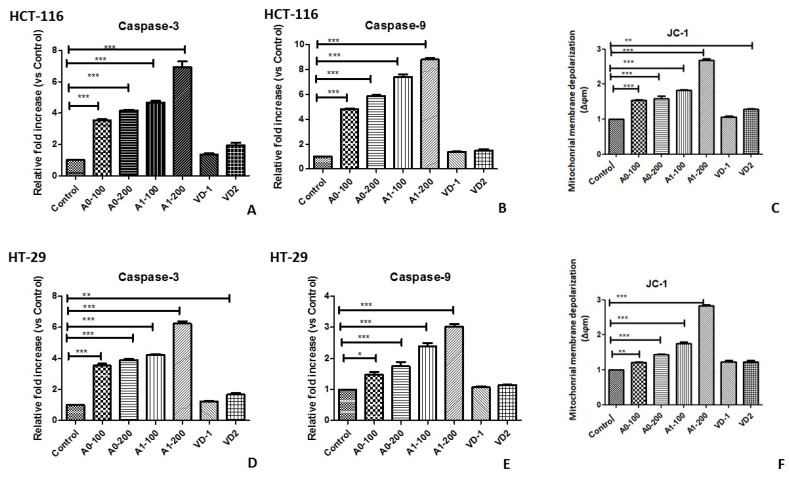
Induction of apoptotic mechanisms by aerogel composite carriers in HT-29. (**A**) Caspase 3 enzyme activation. (**B**) Caspase 9 enzyme activation. (**C**) Mitochondrial membrane depolarization. ** *p* < 0.01, *** *p* < 0.001 compared to the control and HCT-116. (**D**) Caspase 3 enzyme activation. (**E**) Caspase 9 enzyme activation. (**F**) Mitochondrial membrane depolarization * *p* < 0.05, ** *p* < 0.01, *** *p* < 0.001 compared to the control.

As a result of the activation of the caspases, it was determined that the aerogels induced apoptosis in colon cancer cells. As seen in Figure 4A,D, the Aero0 and Aero1 carrier systems were found to increase caspase 3 enzyme activation in colon cancer cells in a dose-dependent manner (*p* < 0.001 and *p* < 0.001, respectively). Moreover, it was also determined that the Aero1 carrier system containing VD further enhanced this activation compared to Aero0. Similarly, caspase 9 enzyme activation increased with increasing doses of Aero0 and Aero1 (*p* < 0.001); although VD caused an increase compared to the control, which was not statistically significant (*p* > 0.05) (Figure 4B,D). However, no change in caspase 8 activity was observed. These data suggest that composite aerogels caused cell death by triggering the internal signaling pathway of the apoptotic mechanism in both colon cancer cells.

The JC-1 staining method was also employed to evaluate cell apoptosis. JC-1 is a fluorescent dye that accumulates in the mitochondrial matrix of healthy cells and produces red fluorescence. However, if MMP is decreased, JC-1 is unable to accumulate in the matrix and instead exists as a monomer, producing green fluorescence. As seen in Figure 4C,F, the change in the JC-1 fluorescent color from red to green with increasing concentrations of composite aerogels suggests that MMP decreases with increasing aerogel concentrations. This decrease was also statistically significant (*p* < 0.001).

In line with our results, Varghese et al. reported that VD triggered apoptosis by increasing caspase 3 activity in HCT-116 cells [60]. Moreover, another study supporting our results reported that VD loaded on nanoparticles induced apoptosis by increasing caspase 3 and caspase 9 enzyme activities in HCT-116 and HT-29 cells [61]. After binding VD to the VDR in colon cancer cells, intracellular calcium levels increased and then activated calmodulin (CAM), dependent kinase II (CAMKII) proteins, and Bcl-2-associated X (BAX) proteins, respectively. Thus, VD triggers mitochondrial damage. As a result of the decreasing MMP, the membrane depolarizes, and the cytochrome C enzyme leaks from the mitochondrial membrane into the cytosol triggering apoptosis by activating caspase 3.

**Figure 5:**
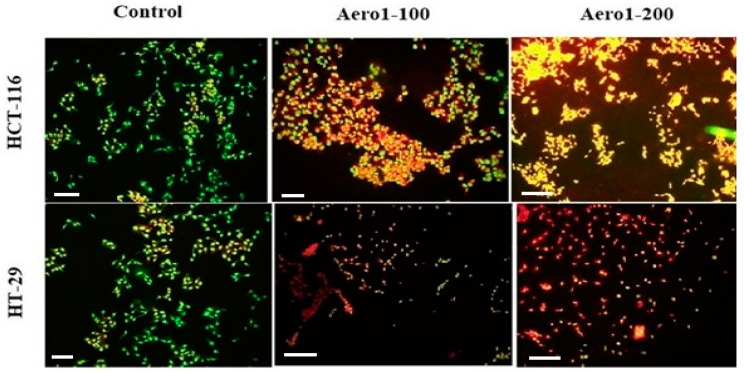
Determination of the dose-dependent apoptotic effects of Aero1 (100 µg/mL and 200 µg/mL) on HT-29 and HCT-116 by acridine orange staining. The scale bar represents 10 µm.

The induction of cell apoptosis by composite aerogels was also demonstrated through morphological visualization using acridine orange staining (Figure 5). This study’s result revealed that the control group cells were stained green, while the Aero1 composite system induced apoptosis in both colon cancer cells, leading to a shift in staining from orange to red. Furthermore, a more pronounced effect was observed in HCT-116 cells. The presence of small and shrunken cells stained orange-red indicated a dose-dependent increase in apoptosis.

### 2.4. Anti-Migratory Effects of Aerogels

In order to examine the effect of aerogels on the migration of colon cancer cells, the “wound healing” assay was employed. The scratch assay is a widely utilized method for investigating cell migration in vitro. This technique is based on the observation that when a new artificial cavity, referred to as a “scratch”, is introduced to a confluent cell monolayer, cells located at the edge of the newly created cavity will migrate toward the opening to close the “scratch” and restore cell–cell contacts. To conduct this assay, the first step involves creating a scratch on a monolayer of cells. Subsequently, the process of cell migration toward the scratch is monitored by capturing images at regular intervals. The obtained images are then analyzed and compared to determine the rate of cell migration in response to the scratch. 

**Figure 6:**
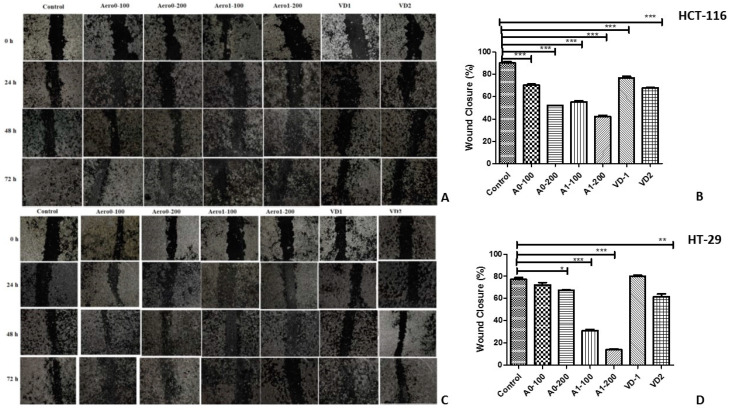
Composite aerogels loaded with VD inhibit colon cancer cell migration. HCT-116 (**A**,**B**) and HT-29 (**C**,**D**) cells were scratched using a 200 μL tip, and the wound widths were recorded at 0, 24, 48, and 72 h. The width of the gaps in three experiments was measured and the means and their standard errors (SEM) were presented in bar graphs using ImageJ software, and the data were analyzed using Prism 5.0. * *p* < 0.05, ** *p* < 0.01, *** *p* < 0.001 compared to the control.

Results of the wound healing assay showed that Aero0, Aero1, and VD inhibited the cell migration in a dose-dependent manner in HCT-116 cells (Figure 6A,B) (*p* < 0.001). It was also observed that the treatment of Aero1 resulted in a significant disruption of intercellular junctions and a decrease in cell size. These changes in cellular morphology were substantial and noteworthy. When the effects of aerogels on HT-29 cell migration were examined, it was determined that Aero1 significantly inhibited cell migration in a dose-dependent manner, as demonstrated by data presented in Figure 6C,D. Furthermore, the inhibitory effect of the Aero1-200 aerogel was particularly pronounced.

The results suggest that the ACNF-VD-containing composite aerogel DDS, serving as a VD carrier platform, exhibits antiproliferative, anti-migratory, and apoptotic effects on HT-29 and HCT-116 colon cancer cell lines. The platform demonstrated a high biocompatibility rate with healthy cells, even at increased doses. The findings from our study will be valuable for the development of new strategies in cancer treatment and provide information for the production of other drug delivery systems.

## 3. Conclusions

The problems related to the absorption and bioavailability of VD in the body and the occurrence of cytotoxic effects, like hypercalcemia, limit its clinical use. Although carbon nanofibers (CNFs) are considered potential drug delivery systems due to their unique properties, they have not exhibited the desired biocompatibility on healthy cells. In our study, composite alginate aerogels were prepared as a result of the cytotoxic effect of CNFs loaded with VD on healthy cells. Sodium alginate is a relatively low-cost material that is often preferred in the development of drug delivery systems due to its low toxicity and high biocompatibility.

Despite its mechanically rigid and stable structure, sodium alginate possesses the ability to adhere to the cell surface, bind to biomolecules, and release them. Sodium alginate aerogels have demonstrated the successful trapping and release of hydrophobic molecules, particularly in aqueous solutions at room temperature. It is believed that these aerogels release their contents due to the pH difference within cells and the action of lysosomal enzymes.

**Figure 7:**
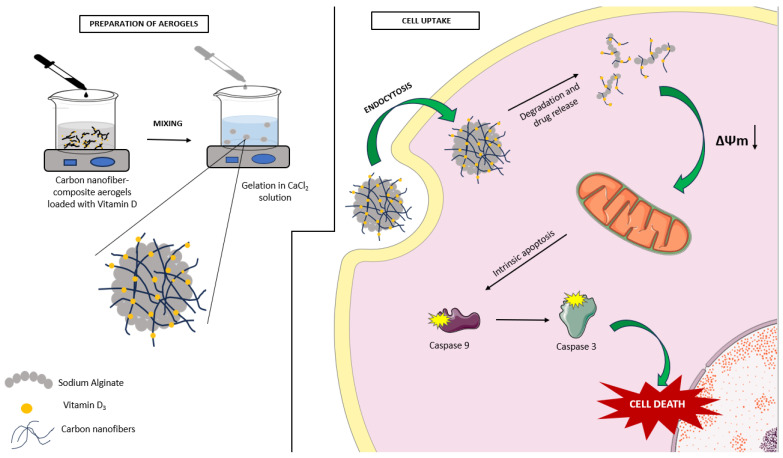
A schematic representation of the effect of vitamin D-loaded CNF sodium alginate conjugate aerogel (Aero1) on human colon cancer cells.

The results of our study indicate that the composite aerogel loaded with VD, referred to as Aero1, not only enhances the antiproliferative activity but also enables a reduction in the required dosage, thereby increasing the bioavailability of VD. Additionally, the study demonstrated that the encapsulation of VD within a nanovehicle can induce apoptosis in human colon cancer cells by activating internal signaling pathways (Figure 7). In conclusion, the nanoencapsulation of VD within an alginate matrix may offer a novel and effective administration strategy that addresses the current limitations of VD, such as its low bioavailability. The development and optimization of targeted, biocompatible, and cost-effective VD delivery systems hold significant potential in preventing a wide range of diseases, including cancer, cardiovascular disease, diabetes, liver disease, renal disease, respiratory disorders, and neurological disorders resulting from VD deficiency.

## 4. Materials and Methods

### 4.1. Reagents

CNFs are supplied by Sigma (>98% carbon basis). Vitamin D3 crystallization is provided by Zhejiang Garden Biochemical High-Tech Co., Ltd. Dulbecco’s Modified Eagle Medium/F12 (DMEM/F12) was purchased from PAN Biotech (Aidenbach, Germany). DMEM medium and fetal bovine serum (FBS) were purchased from Gibco, Thermo Fisher Scientific, Waltham, MA, USA. The MTT Cell Proliferation Assay Kit is provided by Thermo Fisher Scientific, USA. All other chemicals were purchased from Sigma-Aldrich, UK. All the chemicals were of analytically pure grade.

### 4.2. Preparation of Vitamin D Solution

A total of 50 mg of VD was dissolved in 100 mL of 99.9% ethanol, resulting in a concentration of 5 mg/L.

### 4.3. VD Loading and Preparation of Aerogels

The conical-shaped carbon nanofibers (CNFs) have been investigated as a green alternative. Previous studies have demonstrated the efficient adsorption capability of CNFs for organic molecules in a stimulated gastric and intestinal medium [66]. The properties and characterization of CNFs have been published elsewhere, where it was reported that the CNFs used in this study are pyrolitically stripped, with an average diameter of 130 nm, an average pore volume of 0.12 cm^3^/g, an average pore diameter of 89.3 Å, and an average specific surface area of 54 m^2^/g, with a pHPZC value of 4.9 [43]. 

The washing procedure was carried out with 2 L of deionized (DI) water for every 2 g of CNFs using a magnetic stirrer. The CNFs were dried at 85 °C for 24 h after washing. To prepare activated carbon nanofibers (ACNFs), 3 g of CNFs were stirred in 50 mL of 85% H_3_PO_4_ (phosphoric acid) for 24 h and then activated at 250 °C. The resulting ACNFs were diluted with DI water and centrifuged to neutralize the acid value and then dried at 85 °C.

The batch adsorption method was used for the loading of VD on the ACNF. To load vitamin D (VD) onto ACNFs, a dispersion of 150 mg ACNF was prepared in 100 mL of DI water, while 50 mg of VD was dissolved in 100 mL of 99.9% ethanol, resulting in a total volume of 200 mL, and then the two solutions were stirred together to load VD onto the ACNFs. The resulting dispersion was left to incubate at 37 °C in a shaking water bath for 24 h, and then washed with DI water and filtered. The ACNF-VD samples on the filter were dried at 37 °C.

After loading VD onto the ACNF, aerogels were prepared using these ACNF-VD samples. To prepare aerogels, 4 mg of ACNF-VD was stirred in 10 mL of DI water to prepare a 0.4 mg/mL ACNF-VD dispersion, while the ACNF was stirred in 10 mL of DI water to prepare a 0.4 mg/mL ACNF dispersion. The two dispersions were then dispersed with a sonicator. The ACNF-VD dispersion was added dropwise into 10 mL of sodium alginate solution (2 g/100 mL DI water), and after stirring for 10 min with a magnetic stirrer, it was added dropwise into 3 g of CaCl_2_/100 mL DI water solution and left for 24 h. The same method was repeated for the ACNF dispersion. After washing with distilled water, the resulting aerogels were kept at room temperature for 24 h and then dried at 37 °C.

Adsorption quantity (q) was calculated using the data collected from adsorption studies. The light absorption of VD solutions was measured using a UV-visible spectrophotometer before and after the adsorption studies. Initial and final VD concentrations were calculated using the calibration curves prepared previously. The adsorption experiment showed that 1 g of CNF adsorbed 218.57 mg of VD, as calculated using Equation (1) given below.

q = ((C_0_ − C) × V)/m (1)

C_0_: initial concentration of VD; C: final concentration of VD; V: volume of the VD solution; and m: mass of the ACNF.

Two different doses of Aero1 samples were used for the cell culture experiments, which contained 100 µg and 200 µg, respectively, and corresponded to 14 IU and 28 IU of VD, respectively. The following samples were prepared in this study:

- Vitamin D: 1 µM (VD 1);
- Vitamin D: 2 µM (VD 2);
- ACNF (Activated carbon nanofibers) (150 mg) − VD (50 mg) (ACNF − VD);
- Aerogel (sodium alginate) (200 mg) + ACNF (4 mg) (Aero0);
- Aerogel (sodium alginate) (200 mg) + ACNF − VD (4 mg) (Aero1).

### 4.4. Measurements and Characterization of Aerogels

VD batch adsorption studies were conducted using a UV-visible spectrophotometer (Shimadzu 1700) to record the UV spectra of the samples. The morphology of the prepared samples was characterized using Scanning Electron Microscopy (SEM) (Zeiss EVO MA 10, Zeiss). Prior to imaging, the nanoparticles were coated with gold palladium by spraying them with a Quorum SC7620 Mini Spray for 180 s. The applied acceleration voltage was 10 kV. A Fourier-transform infrared spectroscopy (FTIR) analysis was performed using an FT/IR 4700 (Jasco, Tokyo, Japan) spectrometer to examine the bonding structures and functional groups of ACNF, VD, ACNF-VD, Aero0, and VD-loaded Aero1 aerogels. The measurements were taken at an average resolution of 4 cm^−1^ at 4000−450 cm^−1^. XRD studies were carried out using an XRD-6100 (Shimadzu, Japan). XRD patterns of samples were analyzed using a Cu source (λ = 1.54060 Å) over a 2θ range of 5°–100°. The samples were scanned at a 40 kV and 30 mA operating voltage and current, respectively.

### 4.5. Cell Culture Studies

Human colorectal cancer cell lines, HT-29 (ATCC, HTB-38) and HCT-116 (ATCC, CCL-247), were used in this study to investigate the effects of aerogels. HT-29 and HCT-116 cells were cultured in a DMEM medium supplemented with 10% FBS and 1% penicillin-streptomycin at 37 °C and 5% CO_2_ atmosphere. Non-tumorigenic mouse embryonic fibroblast cells NIH/3T3 were cultured in a DMEM/F12 medium containing 10% FBS and 1% penicillin-streptomycin at 37 °C and 5% CO_2_ atmosphere.

### 4.6. Assessment of Cell Viability of Aerogels

The MTT (3-(4,5-dimethylthiazol-2-yl)-2,5-diphentyltetrazolium bromide) method is used to determine the antiproliferative effects of aerogel drug delivery systems (DDSs) on HT-29 and HCT-116 cancer cells and cytotoxic effects on NIH/3T3 cells [67]. Briefly, the cells (1 × 104 cells per well) were seeded in 96-well plates and cultured for 24 h. The next day, the cells were treated with different concentrations of aerogels for 24 h. After incubation, 10 µL MTT solution was added, and the cells were incubated in a CO_2_ incubator for 4 h. The optical density (OD) was read at 570 nm using 630 nm as reference wavelength on a multiwell plate reader (Biotech Instruments, Winooksi, VT, USA). All experiments were repeated twice, and each treatment was run in triplicate. 

The percentage of cell viability was calculated using the equation:

[mean (OD) of treated cells/mean OD of control cells] × 100

### 4.7. Monitoring Cell Proliferation Using the Xcelligence Real-Time Cell Analysis (RTCA)

The xCELLigence RTCA (ACEA Biosciences, Santa Clara, CA, USA) is a non-invasive and impedance-based biosensor system that can measure cell growth, viability, migration, and proliferation. Alterations in cell morphology are continuously monitored in real-time using microelectronics located in the wells of RTCA E-plates. The antiproliferative effect of aerogels was also monitored using an xCELLigence RTCA system. First, 100 μL of cell culture medium was added to each well of an E-plate 16. At approximately 24 h after incubation, the cells were treated with different concentrations of aerogels, and replicated three times. The experiments were run for about 120 h. At the end of the experiment, the growth curves of colorectal cancer cell lines were established according to the cellular density at seeding using cell impedance measurements with the xCELLigence RTCA system, and the IC50 values were calculated using the integrated software of the system.

### 4.8. Apoptotic Potential of Aerogels

#### 4.8.1. Measurement of Caspase Enzyme Activitiy

Caspase 3, 8, and 9 activities were measured with commercial kits (Millipore Sigma, Burlington, MA, USA) according to the manufacturer’s protocols, as described in a previous study [68]. 

#### 4.8.2. Determination of Mitochondrial Membrane Potential (MMP)

The changes in mitochondrial membrane potential (MMP) of colon cancer cells were determined using the JC-1 mitochondrial membrane potential kit (MitoPT JC-1, ImmunoChemistry Technologies, LLC, Davis, CA, USA). Briefly, the cells (1 × 10^6^) were seeded and treated with Aero0 and Aero1 drug delivery systems. After 24 h of incubation, they were collected by centrifugation at 1000 rpm for 10 min. Then, the cells were resuspended in a working solution containing JC1 dye and incubated in a CO_2_ incubator for 15 min. After the incubation, the cells were washed three times, and the plate was read at 510 and 580 nm wavelengths using a fluorescence ELISA reader. Ultimately, 585/510 values were calculated to determine the changes in MMP.

#### 4.8.3. Estimation of Apoptosis by Acridine Orange and Ethidium Bromide Staining

The cells (1 × 10^6^) were seeded and treated with Aero0 and Aero1. The cells were incubated for 24 h. The next day, the cells were washed with sterile PBS and fixed with methanol 70%. Then, the cells were incubated at room temperature for 5 min with acridine orange (100 mg/mL) and ethidium bromide (100 mg/mL). After staining, the cells were washed with PBS and examined under a fluorescent microscope.

### 4.9. Cell Migration Analysis of Aerogels

The migration of HT-29 and HCT-116 cells was evaluated using a scratch wound assay. In brief, 5 × 10^5^ cells/mL were added to 6-well culture plates, and the cells were incubated in a 1% *v*/*v* FBS medium for 24 h to induce starvation. The next day, a cross was made in the middle of each well using 200 μL sterile pipette tips to create a wound. The cells were then washed twice with sterile PBS and a fresh medium containing various concentrations of aerogels (100 and 200 μg/mL), and 1–2 μM of vitamin D was added. The cells were then incubated for 24, 48, and 72 h. Cell migration around the wound was visualized and imaged under an inverted microscope (4× objective lens) with two fields of view per replicate for two replicates. The percentage of wound closure was analyzed using the ImageJ program. We calculated the percentage of wound closure as follows:

Wound closure (%)= ((A_0_ − A_T_)/A_0_) × 100

A_0_ is the initial wound area and A_t_ is the wound area after n hours of the initial scratch.

### 4.10. Statistical Analysis

All data were expressed as the mean and standard error of the mean, derived from the repetition of each test at least three times. Statistical analysis was performed using the GraphPad Prism 5.0 program, where an analysis of variance (ANOVA) was conducted to investigate the effect of the variables. *p*-values of less than 0.05 were considered statistically significant.

## Data Availability

Not Applicable.
